# Short-wavelength light beam *in situ* monitoring growth of InGaN/GaN green LEDs by MOCVD

**DOI:** 10.1186/1556-276X-7-282

**Published:** 2012-05-31

**Authors:** Xiaojuan Sun, Dabing Li, Hang Song, Yiren Chen, Hong Jiang, Guoqing Miao, Zhiming Li

**Affiliations:** 1 State Key Laboratory of Luminescence and Applications, Changchun Institute of Optics, Fine Mechanics and Physics, Chinese Academy of Sciences, 3888 Dongnanhu Road, Changchun, 130033, Peoples' Republic of China; 2 Graduate University of the Chinese Academy of Sciences, Beijing, 100039, Peoples' Republic of China

**Keywords:** InGaN/GaN, Green LED, MOCVD, *in situ* monitoring

## Abstract

In this paper, five-period InGaN/GaN multiple quantum well green light-emitting diodes (LEDs) were grown by metal organic chemical vapor deposition with 405-nm light beam *in situ* monitoring system. Based on the signal of 405-nm *in situ* monitoring system, the related information of growth rate, indium composition and interfacial quality of each InGaN/GaN QW were obtained, and thus, the growth conditions and structural parameters were optimized to grow high-quality InGaN/GaN green LED structure. Finally, a green LED with a wavelength of 509 nm was fabricated under the optimal parameters, which was also proved by ex *situ* characterization such as high-resolution X-ray diffraction, photoluminescence, and electroluminescence. The results demonstrated that short-wavelength *in situ* monitoring system was a quick and non-destroyed tool to provide the growth information on InGaN/GaN, which would accelerate the research and development of GaN-based green LEDs.

## Background

The anticipated high commercial demand for solid-state light and high quality outdoor display applications has significantly accelerated the development of green light-emitting diodes (LEDs). However, the efficiency of green LEDs is still far away from the expectation due to the challenges of high-quality growth of InGaN/GaN multiple quantum wells (MQWs) [1-8]. Compared to the blue LEDs, growing InGaN/GaN MQWs is more complex and difficult since more indium content is required in the active layer for green emissions and relatively lower growth temperature. Moreover, due to the low miscibility of InN in GaN, high volatility of InN and the low thermal decompositional efficiency of ammonia (NH_3_) at low temperature, indium separation, and roughness interface usually exist in high In-content InGaN/GaN MQWs [9,10]. Furthermore, the defects and compressive strain in the InGaN well further decrease the optical transformation of InGaN/GaN MQW LEDs, especially for green LED [11-15]. Besides the growth challenge, the lack of direct *in situ* monitoring system for InGaN/GaN MQWs' growth is another important factor to hamper obtaining high-quality, high-In-content InGaN/GaN MQWs. Generally, the InGaN/GaN MQW LEDs were characterized by ex *situ* tools, e.g., high-resolution X-ray diffraction (HR-XRD), transmission electron microscopy, and scanning electron microscopy, to evaluate their structural and interfacial qualities, which are not powerful tools for production because they are time-consuming and are destroyed. Therefore, the *in situ* monitoring system is required to monitor the whole growth process and provide the related information on growth rate and interfacial quality.

The traditional reflectometers with 950 and/or 633 nm usually are not very helpful to monitor InGaN/GaN MQWs' growth due to no obvious intensity modulation of the reflected light. According to Fabry-Perot oscillation, the phase and the amplitude of oscillations depend on the wavelength of the incident light as well as the thickness of the growing layer and optical constants of the materials. Also, the maximum reflectance occurs if the multiplication of the refractive index and layer thickness is equal to the even number of the half incident light wavelength. Thus, for InGaN/GaN MQWs' growth, phase difference and constructive or destructive interference hardly occur when the incoming light is 950 or 633 nm due to both the low transparent InGaN and thin InGaN/GaN MQWs' layer. Instead, the *in situ* monitoring system with short wavelength can show an intensity modulation, related to interference effects.

In this paper, five-period InGaN/GaN MQW green LEDs were grown by metal organic chemical vapor deposition (MOCVD) with 405-nm light beam *in situ* monitoring system. With the direct and precise monitoring system, the optimized growth condition was obtained easily and high-quality green LEDs with 509 nm were fabricated.

## Methods

**Figure 1:**
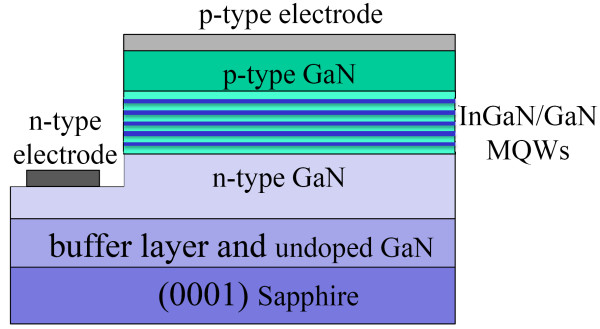
Schematic structure of InGaN/GaN MQW LEDs.

The InGaN/GaN MQW green LEDs were grown on c-plane sapphire substrates by MOCVD with 405-nm light beam *in situ* monitoring system. Trimethylgallium/triethylgallium (TMGa/TEGa), trimethylindium (TMIn), and NH_3_ were the precursors of gallium, indium, and nitrogen, respectively. Silane and biscyclopentadienyl magnesium were used as the n- and p-type dopants, respectively. The growth process was as follows: firstly, the substrate was thermally cleaned at 1,050 °C for 10 min before a 25-nm-thick low-temperature (LT)-GaN buffer layer was deposited at 500 °C. After the growth of LT-GaN buffer layer, the growth temperature was ramped to 1,010 °C, and a 2-μm-thick undoped GaN and 1-μm-thick Si-doped GaN epilayers were grown in sequence. Then, the growth temperature was decreased to grow InGaN/GaN MQWs. For the InGaN/GaN MQWs' growth, a modulated temperature growth mode was employed, that is, the growth temperature for GaN barrier and InGaN well layers was different. Here, the growth temperature for barrier and well layers were 780 °C and 650 °C, respectively. Finally, a p-GaN layer was deposited. The schematic InGaN/GaN green LED structure was shown in Figure 1.

In order to obtain high-quality InGaN/GaN MQWs, the parameters for InGaN/GaN growth were optimized based on the *in situ* monitoring curve. For *in situ* monitoring system, the incident light is partly reflected from the epilayer surface; another partly penetrates into the epilayer and is reflected at the interface between the epilayer and substrate and then travels back to the epilayer surface leading to the intensity modulation of the reflected light. The maximum reflectance occurs when the path difference is equal to the even number of half wavelength:

(1) 2nd=mλ

where *n* is the refractive index; *d*, thickness of epilayer; *m*, even number; and *λ*, incident light wavelength. Considering the relationship between the thickness of epilayer and the growth rate:

(2) d=r×t

where *r* is the growth rate of the epilayer, and *t* is the growth time. Then, the growth rate can be estimated from the reflectance of *in situ* monitoring system:

(3) $$r=\frac{m\lambda }{2nt}$$

Furthermore, the amplitude of the reflectance increases with the increase of the In content, and the intensity of the reflectance damps more and more with the interface of GaN and InGaN becoming rougher and rougher. Thus, the information of In composition and interface morphology of InGaN/GaN MQWs can be obtained from the reflectance of *in situ* monitoring system.

To activate the Mg-doped GaN, the samples were annealed by rapid thermal annealing at 750 °C for 10 min. HR-XRD and photoluminescence (PL) were employed to characterize structural and optical properties of InGaN/GaN MQWs.

## Results and discussion

**Figure 2:**
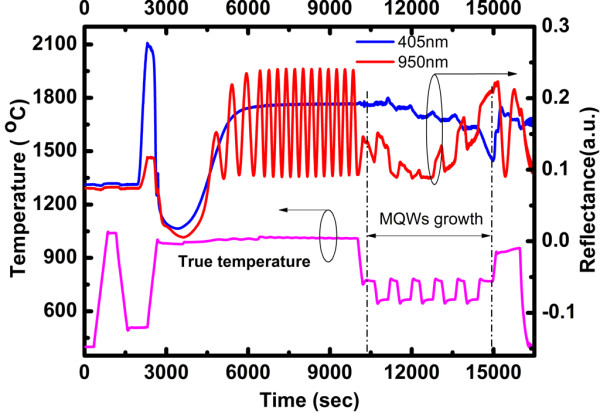
Reflectance traces of 950 and 405 nm and true temperature transients for InGaN/GaN MQW LEDs.

Figure 2 shows the reflectance traces for 950 and 405 nm and true temperature transients for typical InGaN/GaN MQW LEDs' growth. As can be seen, the 950-nm light beam *in situ* monitoring system can still be used for GaN growth analysis, while it becomes unpowerful for InGaN/GaN MQWs evolution. However, the 405-nm signal is sensitive to InGaN/GaN quantum wells and independent on the GaN underlayer due to the GaN layers absorbing the light at 405 nm and causing no further oscillations. The relationship between the bandgap and temperature is as follows [16]:

(4) $$E_{g}=E_{g}(0)-7.7\times 10^{-4}\times \frac{T^{2}}{T+600}$$

Since the growth temperature for the GaN underlayer is 1,010 °C, the bandgap of GaN is 2.797 eV. Then, the absorbing wavelength is correspondingly 443 nm. Thus, the bandgap of GaN at 1,010 °C is narrow enough to absorb the light with a wavelength of 405 nm. With increasing the layer thickness, the intensity maxima and minima approach the constant value of reflectance characteristic for the layer surface, as shown in Figure 2. Then, the growth of InGaN quantum wells on top of the GaN buffer can be studied in detail. Obviously, differently from the 950-nm reflectance, the 405-nm data taken during buffer layer growth and MQWs growth are no longer correlated, which indicate that small deviations in the GaN growth rate do not limit the quantitative analysis of the 405-nm data measured during the MQWs growth.

It can also be seen that for the 405-nm data, the quantum wells and barriers are distinguished due to the refractive index less sensitive to temperature variations at 405 nm than at 633 nm. Thus, the information of wells and barriers' growth rate, thickness, and interface roughness can be obtained by fitting or comparing the 405-light beam *in situ* monitoring curves, which is the direct evidence for optimizing InGaN/GaN MQWs' growing conditions and investigating the complex evolution of InGaN/GaN MQWs. Furthermore, the amplitude of the 405-nm reflectance oscillation enhances with the increased of indium content, resulting from the changed of refractive index *n*. Besides, it is known that the strain effect between GaN barrier layer and InGaN well layer can also cause the change of refractive index. Therefore, the InGaN composition as well as the strain effect can be derived from the amplitude of Febry-Perot oscillation in the measured 405-nm reflectance transient.

**Figure 3:**
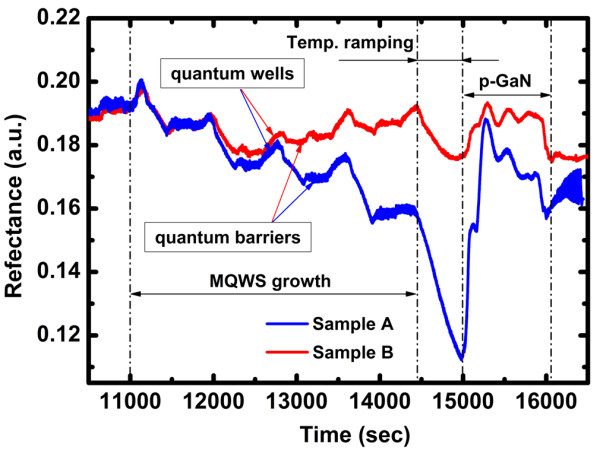
**Reflectance traces of InGaN/GaN MQW green LEDs' growth monitored by 405-nm*in situ*system.**

Figure 3 displays two typical reflectance traces of InGaN/GaN MQW green LEDs. As can be seen, all the wells and barriers are clearly distinguished since the 405-nm *in situ* reflectance signal is not sensitive to temperature changes between wells and barriers' growth. The blue curve shows the data for a nonideal InGaN/GaN MQWs' growth (sample A). It displays the reflectance oscillation damping, indicating the interface between wells and barriers becoming rougher and rougher. As known, the growth of InGaN/GaN is very complex and sensitive to the growth condition, especially the growth temperature. Due to the high volatility of InN, the high In composition of InGaN/GaN MQWs should be grown at low temperature. However, the lower growth temperature results in a poor crystalline quality of GaN barrier attributed to the low surface mobility of adatoms in the low growth temperature as well as the increase of nitrogen vacancy due to the low cracking efficiency of ammonia.

As mentioned above, variations of the indium content as well as of the morphology of the GaN and InGaN layers influence the 405-nm reflectance transients during MQWs' growth. In addition, the InGaN growth rate can also modulate the oscillation character of 405-nm reflectance signal due to the change of interference beam path difference. Thus, the growth information can be directly derived from the 405-nm light beam *in situ* reflectance traces. For high In-content InGaN/GaN MQWs, an extremely high V/III ratio is needed to conquer the nitrogen deficiency on the growing surface [17]. However, an optimized flow rate of TMGa and TMIn is also required to obtain high In composition. Using 405-nm short-wavelength light beam *in situ* monitoring system, the optimized source flow rate can be easily obtained. Furthermore, the 405-nm signal gives the direct evidence that rather than the In flow rate, the growth temperature influences the In content of InGaN well layer deeply. The red reflectance trace in Figure 3 shows the optimized growth data for sample B.

**Figure 4:**
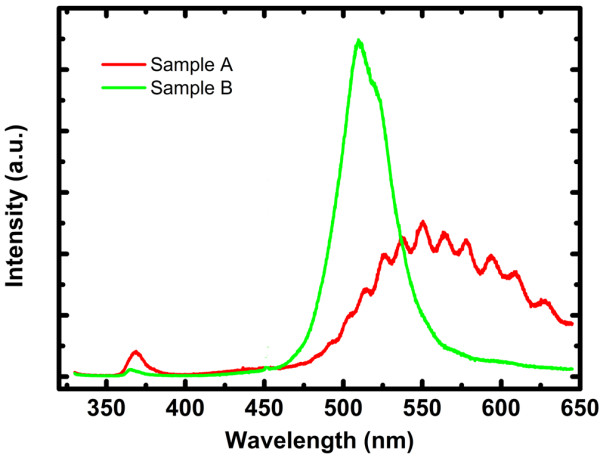
PL spectra for the samples A and B.

To further confirm the quality of the samples growth monitored by 405-nm short-wavelength light beam *in situ* system, the room temperature PL and HR-XRD have also been studied. Figure 4 exhibits the PL spectra of the two InGaN/GaN MQW LEDs before and after optimization according to the 405-nm signal. It has been shown that for the nonideal growth sample A, the PL peak wavelength displayed poor optical property with a strong yellow luminescence, caused by impurities or defects, and nearly no obvious green luminescence can be seen. Otherwise, the optimized sample B shows strong green luminescence about 509 nm, indicating the InGaN/GaN MQW green LEDs had a high quality. These results agree with the information gained by the 405-nm signal, meaning the trustworthy of the 405-nm *in situ* monitoring system.

**Figure 5:**
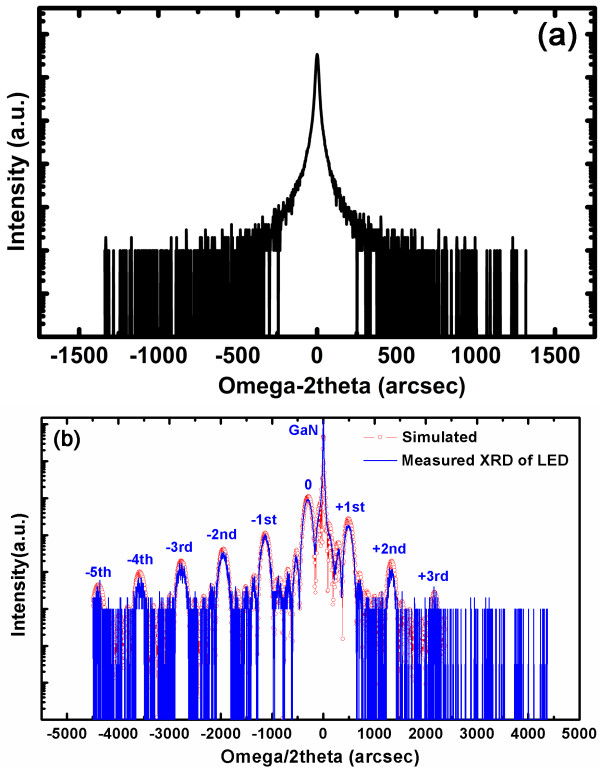
**Measured HR-XRD curves and simulation.** (**a**) Measured HR-XRD curves for sample A, and (**b**) measured HRXRD curves and its simulation for sample B.

Figure 5a shows the HR-XRD curves for samples A and B. For sample B, five numbers of satellite peaks are clearly observed, but no satellite peak is observed for sample A, which suggests that the interface of sample B is flatter than that of sample A due to the satellite peaks reduced by the roughness interface for MQWs' structure. Meanwhile, for the sample B, the fringe peaks (secondary satellite peaks) between satellite peaks can be also observed, which means the MQWs' quality is good. According to the numbers of fringe peaks, the total period number could be deduced, that is 3 (fringe numbers) + 2 = 5 (total periods), which is in good agreement with the designed period numbers, further indicating excellent layer periodicity and interface quality for sample B. In order to get more information of the MQWs structure, a simulation was carried out to fit the experimental HR-XRD pattern (Figure 5b). According to the simulation, the thicknesses of well and barrier are 3.0 and 17.0 nm with an error ±0.1, respectively. Furthermore, the In content in the InGaN well layer is 0.21.

**Figure 6:**
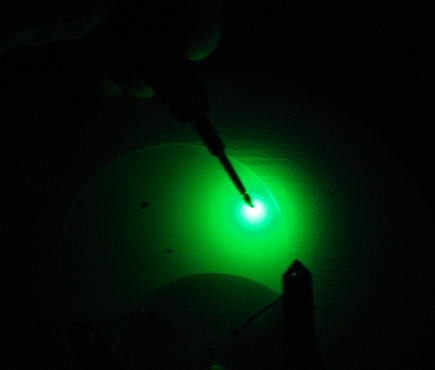
Electroluminescence photo of the optimized InGaN/GaN MQW green LEDs.

Figure 6 displays the electroluminescence character of the sample B with five-period InGaN/GaN MQWs optimized according to the 405-nm *in situ* monitoring system. Obviously, the high brightness green light further confirmed the high quality of InGaN/GaN MQW green LEDs optimized by 405-nm light beam *in situ* monitoring system.

## Conclusions

In summary, five-period InGaN/GaN MQW green LEDs were grown by MOCVD with 405-nm light beam *in situ* monitoring system. The results showed that 405-nm reflectance trace could provide the growth information, such as the interface morphology, the In composition, the growth rate, and so on. Thus, according to the *in situ* 405-nm light monitoring signals, the parameters for growth of high-quality, high In-content InGaN/GaN MQW green LEDs can be optimized easily. The PL spectra and HR-XRD curve and electroluminescence character confirmed the high quality of InGaN/GaN MQW green LEDs optimized by 405-nm *in situ* monitoring data. The results show that the short-wavelength *in situ* monitoring system is a powerful, noninvasive real-time tool for the growth of InGaN/GaN MQWs.

## Abbreviations

HR-XRD, high-resolution X-ray diffraction; LED, light-emitting diode; MOCVD, metal organic chemical vapor deposition; MQWs, multiple quantum wells; NH3, ammonia; PL, photoluminescence; TMGa, trimethylgallium; TMIn, trimethylindium.

## Competing interests

The authors declare that they have no competing interests.

## Authors’ contributions

XS, DL, and HS conceived of the study and participated in its design and drafted the manuscript. XS, ZL, and YC carried out the experiments. HJ and GM guided in the revision of the manuscript. All authors read and approved the final manuscript.

## Authors' information

XS and YC are assistant professors, DL, HS, HJ, and GM are professors, and ZL is an associate professor at the State Key Laboratory of Luminescence and Applications, Changchun Institute of Optics, Fine Mechanics and Physics, Chinese Academy of Sciences, 3888 Dongnanhu Road, Changchun, 130033, Peoples' Republic of China. XS and YC are also affiliated to the Graduate University of the Chinese Academy of Sciences, Beijing, 100039, Peoples' Republic of China.
